# A Three-Tier In Vitro Strategy for Accelerated Pine Breeding and Resistance Research Against Pine Wilt Disease

**DOI:** 10.3390/plants15020246

**Published:** 2026-01-13

**Authors:** Zi-Hui Zhu, Yan-Fei Liao, Yang-Chun-Zi Liao, Hui Sun, Jian-Ren Ye, Li-Hua Zhu

**Affiliations:** 1 College of Forestry and Grassland, Nanjing Forestry University, Nanjing 210037, China; zhuzihui@njfu.edu.cn (Z.-H.Z.); liaoyanfei@njfu.edu.cn (Y.-F.L.); yangchunzi.liao@ut.ee (Y.-C.-Z.L.); hui.sun@njfu.edu.cn (H.S.); jrye@njfu.com.cn (J.-R.Y.); 2 Co-Innovation Center for Sustainable Forestry in Southern China, Nanjing Forestry University, Nanjing 210037, China; 3 Department of Botany, University of Tartu, Ülikooli 18, 50090 Tartu, Estonia; 4 Department of Forest Sciences, University of Helsinki, P.O. Box 27, FI-00014 Helsinki, Finland

**Keywords:** pine wilt disease, *Bursaphelenchus xylophilus*, in vitro screening, somatic embryogenesis, resistance breeding, model system

## Abstract

Pine wilt disease (PWD), caused by the pine wood nematode (PWN) *Bursaphelenchus xylophilus*, is a globally destructive threat to coniferous forests, causing severe ecological and economic losses. Conventional resistance breeding is critically hampered by long life cycles of trees and field evaluation challenges. To address these limitations, we developed a three-tier biotechnology pipeline with a dual-output goal (generating both resistant germplasm and mechanistic insights) designed to bridge the in vitro–field gap. This strategy is founded upon the resolution of a longstanding pathogenesis debate, which established aseptic PWNs as a standardized research tool. The pipeline integrates high-throughput in vitro cellular screening (Tier 1), whole-plant validation via organogenesis (Tier 2), and scaled production coupled with mechanistic investigation through somatic embryogenesis (Tier 3). Tier 1 enables rapid phenotypic screening, Tier 2 validates resistance in whole plants, and Tier 3 facilitates mass production and in-depth study. It operates as a closed-loop, knowledge-driven system, simultaneously accelerating PWN-resistant germplasm development and empowering molecular mechanism discovery. Validated across *Pinus massoniana* and *P. densiflora*, this work provides a concrete, community-usable model system that directly addresses a core methodological bottleneck in forest pathology. This strategy effectively bridges the in vitro–field gap, offering a replicable model for perennial crop breeding and contributing to resilient forest management.

## 1. Introduction

Pine wilt disease (PWD), caused by *Bursaphelenchus xylophilus*, is one of the most destructive diseases of coniferous forests, causing extensive tree mortality and severe ecological and economic damage [1]. Since its initial identification in Japan in the early 20th century, PWD has expanded from a regional issue to a global threat, spreading throughout Asia and reaching Europe and Africa [2,3,4]. Its continued emergence in new regions, including Armenia and notably France in 2025 [5,6], underscores its ongoing geographic expansion. Within China, PWD has reached a particularly severe scale, with official 2021 reports documenting its presence in 19 provinces and 742 districts, impacting over 1.72 million hectares and resulting in the loss of approximately 14.08 million pine trees [7]. Most critically, this expansion now directly threatens global climate goals, as pine forests are a major carbon sink. Recent analysis indicates that PWD has already caused a carbon sink deficit of 1857 TgC since 1977, and could threaten up to 78% of Eurasia’s boreal forests by 2100 [8]. The disease primarily affects key conifer species such as *Pinus massoniana*, *P. densiflora*, and *P. thunbergii*, all of which are highly susceptible [8,9]. Recent reviews emphasize the biological complexity of PWD, its management challenges under shifting climate and trade patterns [10], and advances in understanding PWN–host interactions at the molecular level [11].

Conventional breeding for PWD resistance has been hindered by the long life cycles of conifers, logistical difficulties in field inoculations, and seasonal constraints [12,13]. The decade-long timeline from crossing to the deployment of improved stock is mismatched with the rapid spread of the disease, emphasizing the urgent need for accelerated breeding approaches. This methodological gap has been explicitly acknowledged, with recent calls for standardized molecular model systems to address micro-scale knowledge deficits in pathology [14].

Plant biotechnology, particularly tissue culture, provides powerful tools to overcome these constraints. In vitro systems enable rapid clonal propagation, germplasm conservation, and controlled host–pathogen studies, supporting efficient resistance screening [15,16]. However, the effectiveness of any in vitro strategy depends on a clear understanding of disease etiology.

For decades, PWD pathogenesis was contested between in a fundamental scientific controversy, with two main hypotheses: “nematode-autonomous pathogenesis” and “bacteria-essential” [17,18]. This controversy impeded targeted research and strategy development. Through a systematic research program spanning over ten years, our laboratory has resolved this debate. We established a reproducible protocol for generating aseptic PWNs [19] and conclusively demonstrated their autonomous pathogenicity on aseptic pine seedlings [20,21,22] and embryogenic calli [23]. This work enabled not only precise pathogenicity assays but also high-throughput cellular resistance screening and initial mechanistic studies [24]. Settling this foundational question provided the essential validated tool upon which our integrated biotechnology pipeline was built.

Here, we synthesize our work into an integrated, cross-species biotechnology framework. This framework provides a concrete and scalable response to the community’s call for standardized model systems [14]. We describe its validation as interoperable toolkit for accelerated breeding and explore how it bridges high-throughput screening with molecular mechanism discovery. Finally, we discuss key deployment considerations and future integrations with emerging technologies. This three-tier in vitro pipeline is designed with a dual-output purpose: to simultaneously generate resistant breeding material and advance mechanistic knowledge of PWN resistance.

## 2. Resolving a Foundational Debate: Establishing the Aseptic PWN as a Definitive Research Tool

### 2.1. Historical Controversy and Methodological Stalemate

The pathogenesis of PWD has been historically controversial, significantly hindered research progress. Two competing hypotheses emerged: the “bacteria-essential” hypothesis, which posited that nematode-associated bacteria were required for virulence [25,26,27], and the “nematode-autonomous pathogenesis” hypothesis supported by earlier, less stringent in vitro studies [28,29]. This fundamental ambiguity impeded the development of targeted control and consistent experimental approaches.

### 2.2. Definitive Evidence: A Methodological Breakthrough and Systematic Validation

A pivotal advance came from our systematic research, beginning with the establishment of a reproducible protocol for generating and maintaining aseptic PWNs [19]. Using this tool, we demonstrated conclusively that aseptic PWNs consistently induced wilting in aseptic pine seedlings, confirming their autonomous pathogenicity [20,21], a finding later corroborated independently [30]. Furthermore, we expanded this evidence by showing that aseptic PWNs induce necrosis, reduce cell viability, and reproduce on embryogenic callus of multiple pine species [23,24]. This work effectively transferred the PWD pathosystem to a fully defined in vitro platform, eliminating the confounding variables of open environmental systems.

### 2.3. Implication: A Paradigm Solidified for Precision Research

The multi-tiered evidence presented here from undifferentiated callus to whole plants, definitively resolved the historical debate over PWN pathogenesis. This work establishes aseptic PWN as the sole causal agent of PWD and validates their use as an indispensable tool for reductionist research. Building on this foundation, we developed the accelerated breeding strategy described in this review. By establishing the aseptic PWN as a definitive research tool, we have created an essential ‘baseline model’ for the PWD pathosystem. This controlled, binary host–pathogen interaction serves as a critical reference point, enabling systematic investigation into the roles of associated microbes, environmental factors, and host genetic variation. It fulfills a key prerequisite for any molecular model system: a standardized and unambiguous causal agent.

## 3. Overview of In Vitro Approaches in Plant–Pathogen Interactions

In vitro systems have long been used in phytopathology to study host–pathogen interactions in a simplified and controlled environment. The principle that cultured plant cells can express specific resistance or susceptibility traits is well-supported, as demonstrated in diverse systems such as papaya [31], banana [32], and pineapple [33]. For tree species, whose size and longevity present practical challenges, tissue culture is particularly advantageous. This approach has been successfully applied to various tree pathosystems [34], including Dutch elm disease [35] and fire blight [36].

For nematode diseases, the development of aseptic inoculation systems represented a critical advance. Early in vitro studies using callus or micropropagated shoots provided a platform to dissect the roles of PWNs and their associated bacteria [20,26], paving the way for the integration of such systems into resistance breeding programs [37]. Similar in vitro selection approaches have also been employed to assess resistance against fungal pathogens in conifers, such as *Gremmeniella abietina* [38] and *Heterobasidion annosum* [39].

## 4. An Integrated, Cross-Species Biotechnology Pipeline: From Screening to Mechanism

### 4.1. Foundational Framework: A Three-Tiered Pipeline

**Figure 1:**
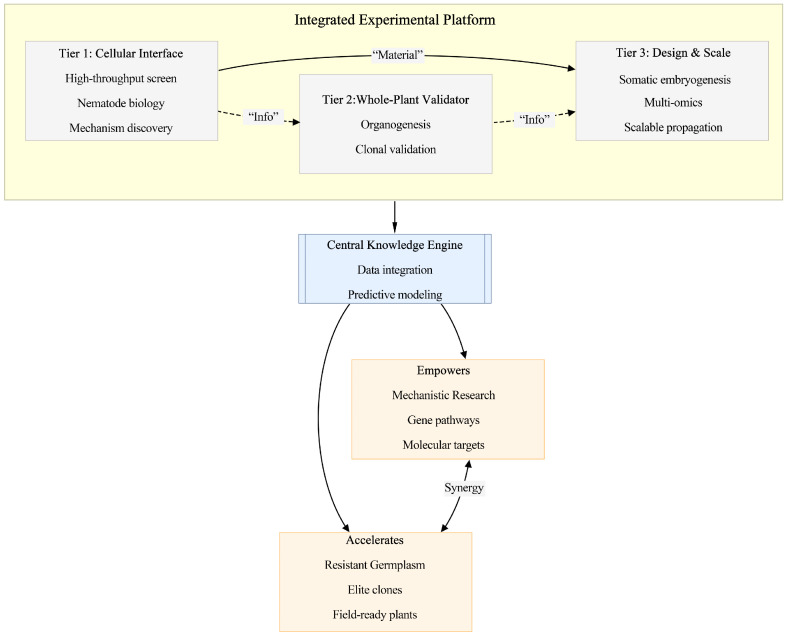
Schematic of the three-tier, dual-output strategy. The Integrated Experimental Platform comprises three interoperable tiers. Tier 1 serves as a versatile cellular interface for high-throughput screening, basic nematode biology, and comparative mechanistic studies. Tier 2 provides a pathway for whole-plant resistance validation via organogenesis from seeds. Tier 3 enables scaled production and in-depth analysis via somatic embryogenesis. Strategic information and material flows connect the tiers (arrows). Data from all tiers converge into a Central Knowledge Engine, creating a closed-loop system that continuously refines the platform. This engine drives two synergistic outputs: empowering mechanistic research and accelerating the development of resistant germplasm, thereby bridging the in vitro–field gap.

We propose a flexible and interoperable biotechnology framework structured into three complementary tiers (Figure 1). This modular design enables deployment independently or in combination, depending on available biological material and research objectives. The detailed media formulations, culture conditions, and inoculation procedures underlying each tier have been described in the cited original studies.

Tier 1: High-Throughput Cellular Screening

This tier serves as the primary high-throughput screening platform. It utilizes callus cultures (embryogenic and non-embryogenic) to study host–pathogen interactions at the cellular level. It serves three primary purposes: (i) non-embryogenic callus provides a simplified model for basic nematode biology; (ii) embryogenic callus enables rapid high-throughput phenotypic screening (within 2–3 weeks) to identify candidate resistant lines for downstream validation; and (iii) it allows direct comparative analysis of resistant and susceptible lines to investigate early cellular and molecular defense mechanisms. Tier 1 thus functions as a multifunctional discovery platform supporting both applied breeding and fundamental research. Because pine wood nematodes feed on living parenchyma cells during early infection, the callus-based system in Tier 1 captures a biologically relevant cellular interface for controlled studies of pine–PWN interactions.

Tier 2: Whole-Plant Validation via Micropropagation

This tier provides a direct and independent route for functional resistance validation. It employs organogenesis (e.g., from cotyledonary nodes) to generate clonal plantlets for direct resistance testing. It enables functional validation of resistance in a whole-plant context within months, significantly compressing the traditional selection cycle.

Tier 3: Scalable Production and In-Depth Mechanistic Analysis

This tier enables the mass production of elite genotypes and supports high-resolution molecular studies. It utilizes somatic embryogenesis to enable mass production of genetically uniform plantlets for field deployment and supports high-resolution molecular studies to elucidate resistance mechanisms.

The three tiers operate synergistically through complementary technological pathways. Tier 1 and Tier 2 function in parallel: Tier 1 uses embryogenic callus for rapid cellular screening, with promising genotypes advanced to Tier 3 for scaling, while Tier 2 uses organogenesis from seeds for efficient whole-plant validation independently of embryogenic culture. This dual-track approach offers flexibility in experimental entry based on material availability (cell lines vs. seeds) and research goals (in-depth analysis vs. broad validation). Both tracks integrate through the shared Central Knowledge Engine (Figure 1), forming a closed-loop system that continuously refines the platform.

### 4.2. Validation in P. massoniana: A Comprehensive Model

Our research on *P. massoniana* provides the most complete in vitro validation of the three-tier framework. Parallel studies in *P. densiflora* independently confirm its effectiveness and contribute important long-term field performance data.

**Figure 2:**
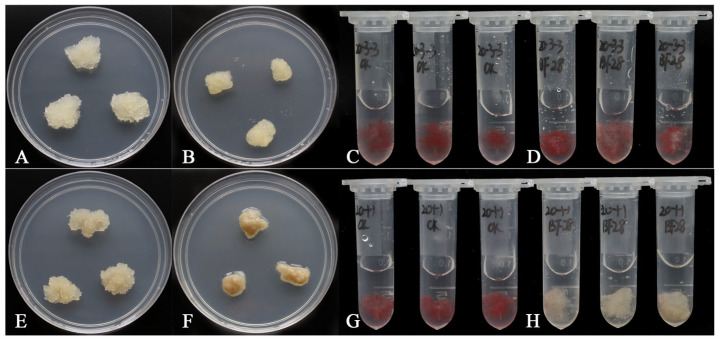
High-throughput in vitro screening for pine wood nematode resistance at the embryogenic callus level. (**A**–**D**) Resistant embryogenic callus line GX20-3-3 at 10 days post-inoculation (dpi). (**A**) Callus inoculated with sterile water maintains a healthy, creamy-yellow appearance. (**B**) Callus inoculated with aseptic *Bursaphelenchus xylophilus* (PWN) shows minimal browning and sustained growth, indicating resistance. (**C**,**D**) Triphenyl tetrazolium chloride (TTC) staining confirms high cell viability post-inoculation, indicated by red coloration reflecting active dehydrogenase activity. (**E**–**H**) Susceptible embryogenic callus line GX20-1-1 at 10 dpi. (**E**) Healthy control callus. (**F**) PWN-inoculated callus shows extensive necrosis, severe browning, and structural collapse. (**G**) TTC staining of control callus shows bright red coloration, indicating active dehydrogenase activity and high cell viability. (**H**) TTC staining reveals a significant loss of dehydrogenase activity, corresponding to the loss of cell viability in PWN-inoculated callus (no coloration). Images adapted from Chen et al. [24] with permission.

Tier 1: An in vitro callus evaluation system was established [23]. Inoculation of embryogenic calli with aseptic PWNs enabled the identification of highly resistant lines (e.g., GX20-3-3), characterized by minimal browning, maintained cell viability, and significantly suppressed nematode multiplication, in contrast to susceptible lines like GX20-1-1 [24] (Figure 2). This cellular assay delivers a preliminary resistance phenotype within 2–3 weeks post-inoculation, positioning Tier 1 as the rapid front end of the accelerated breeding pipeline. These responses align with resistance phenotypes reported in other conifer–pathogen systems, supporting the biological relevance of callus-based assays [38,39].

**Figure 3:**
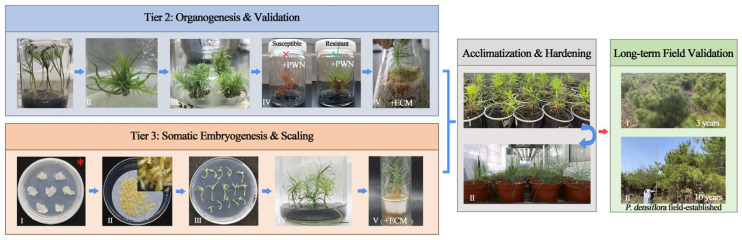
Two parallel and enhanceable in vitro pathways leading to field-validated resistant pines. (Tier 2: Organogenesis & Validation) In vitro identification of PWN-resistant *Pinus massoniana* clones through micropropagation. (**I**) Aseptic seedling, (**II**) cotyledonary node with axillary buds, (**III**) proliferating axillary buds, (**IV**) representative susceptible (×) and resistant (√) clones after challenge with aseptic PWN (+PWN), and (**V**) A plantlet associated with ectomycorrhizal fungi (+ECM). Photographs (**I**–**V**) adapted from [40,41]. (Tier 3: Somatic Embryogenesis & Scaling) Scalable production of plants from resistant embryogenic callus lines (*, from Tier 1 screening) via somatic embryogenesis. (**I**) Embryogenic callus line, (**II**) maturation of somatic embryos, (**III**) germination of somatic embryos, (**IV**) somatic plantlets, and (**V**) a plantlet with ectomycorrhizae (+ECM). Photographs (**I**–**V**) adapted from [42]. (Acclimatization & Hardening) Representative stages of acclimatization for plantlets from both pathways, resulting in hardened plants ready for field trials. Photographs in this panel are previously unpublished data. (Long-term field validation) Field performance of a micropropagated *P. densiflora* clones documented at 3 and 10 years post-establishment. Field images are previously unpublished data.

Tier 2: A robust organogenesis protocol from cotyledonary node explants was developed [40]. Screening of regenerated microshoots revealed significant clonal variation in PWN resistance, leading to the identification of the highly resistant Clone 227, which exhibited 0% wilting rate and suppressed nematode multiplication in shoots by over 90% compared to the most susceptible clone, Clone 253 [40]. Genetic stability of the regenerants was confirmed using SSR markers [40], fulfilling a key requirement for clonal deployment in breeding programs. The entire process—from explant to rooted, nematode-challenged plantlets—can be completed within several months, compressing the traditional year-long selection and propagation. Selected resistant clones can be further enhanced through ectomycorrhizal synthesis with *Pisolithus* spp., as established in our prior work [41]. The complete workflow of this accelerated organogenesis pathway, alongside the parallel somatic embryogenesis route, is summarized in Figure 3.

Tier 3: The somatic embryogenesis process was optimized, with abscisic acid (ABA) and polyethylene glycol (PEG) identified as key factors regulators of somatic embryo maturation and germination [42,43]. Plantlets regenerated from different embryogenic cell lines (ECLs) exhibited varying resistance, with ECL 20-1-7 showing superior performance [43]. Notably, inoculation with the ectomycorrhizal (ECM) fungus *Pisolithus orientalis* significantly increased the acclimatization survival of somatic plantlets (from 37% to 85%) and reduced post-PWN wilting [43], demonstrating how biotic partners can be integrated to overcome acclimatization bottlenecks. For somatic embryogenesis responsive genotypes, this tier provides a direct, scalable route to produce clonally uniform plantlets for advanced mechanistic studies and field deployment, bypassing the slow and unreliable conventional propagation from mature trees.

### 4.3. Parallel Evidence from P. densiflora (Japanese Red Pine)

Research in *P. densiflora* provides independent validation of the pipeline, particularly at Tier 2. A highly efficient micropropagation system via axillary budding from cotyledonary nodes was established [20,37] and employed to evaluate nematode resistance in regenerated microshoots from various seed sources under sterile conditions. This led to the identification of the highly resistant Clone 8–4, which displayed a wilting rate of only 20%—in stark contrast to the most susceptible clone (Clone 6–4) with a 100% rate. Moreover, nematode recovery from Clone 8–4 shoots was reduced by approximately 83% compared to the highest-yielding susceptible clone (Clone 5–10) [37]. Despite its strong resistance, Clone 8–4 showed poor adventitious rooting, limiting its propagation. In contrast, Clone 1-A [20], with moderate resistance, exhibits high propagation rates and robust rooting. Several hundred regenerated plantlets of Clone 1-A have been transplanted into PWD-affected areas, including the Baima Teaching and Research Base of Nanjing Forestry University (Figure 3) and the Xiashu Forest Farm since 2012. Regular monitoring indicated no apparent PWD outbreaks in the experimental plots until at least mid-2019. Access restrictions during the COVID-19 pandemic (2020–2021) prevented field surveys. Upon resuming field inspections at the Baima site, quantitative contrasts were documented across follow-up surveys. In January 2022, susceptible *P. densiflora* seedling controls showed 50% mortality, whereas Clone 1-A plantlets showed 0% mortality. In a subsequent inspection in 2024, susceptible controls reached 100% mortality, whereas Clone 1-A plantlets exhibited substantially lower mortality (31.3%). These natural epidemic observations provide field-based quantitative support that resistance identified in vitro can translate into durable field resilience.

As shown in Table 1, the cross-species validation confirms the effectiveness of the three-tiered biotechnology pipeline for screening and validating resistant pine lines. These results demonstrate that the organogenesis pathway provide a valid and efficient route for PWN resistance screening in a second pine species, and that resistance identified in seedling progeny can be rapidly captured, multiplied, and functionally validated through in vitro regeneration.

Organogenesis thus not only validates PWN resistance but also substantially accelerates traditional breeding. By compressing the multi-year cycle of phenotypic selection and clonal propagation into several months, this represents a core acceleration mechanism within the integrated biotechnology pipeline. Statistical analyses supporting these resistance phenotypes are reported in the cited original studies.

## 5. Critical Considerations and Technological Synergy

### 5.1. Ensuring Genetic Fidelity

The success of micropropagation-based breeding program depends on maintaining genetic stability, yet somaclonal variation remains a risk in prolonged cultures [44]. Continuous genetic monitoring using molecular markers such as RAPD [45,46] or SSRs [47] is therefore essential. Studies in *P. pinaster* [48] and *P. massoniana* [40,42] have generally demonstrated stable embryogenic cultures but underscore the need for ongoing surveillance to preserve clonal fidelity- a critical requirement for commercial forestry. In practice, genetic quality control should be embedded as a routine checkpoint throughout the pipeline, from callus maintenance to elite line multiplication.

### 5.2. Comparing Regeneration Pathways

Both organogenesis [37,40] and somatic embryogenesis [42,43] are viable within this pipeline, each with distinct strategic advantages. Organogenesis is more readily established across genotypes and is efficient for specific clones, making it ideal for Tier 2 validation and smaller-scale production. In contrast, SE, though technically more demanding, offers superior scalability, supports cryopreservation, and is suitable for genetic transformation [49,50]. The choice of pathway can be tailored to program objectives, species, and resources. Hybrid strategies, using organogenesis for rapid initial validation and SE for scaling or gene editing of elite genotypes, may be particularly effective. This framework is intended to complement, rather than replace, conventional progeny testing and emerging genome-enabled breeding approaches by providing standardized, rapid phenotypes and clonally propagated materials.

## 6. Challenges and Future Perspectives

The expanding threat of PWD is propelled by increased global trade and accelerating environmental change. Climate modeling predicts that warming temperatures could expand high-risk areas by over 50% within decades [51], a trend linked to the northward shift of suitable habitats for its primary insect vector [52]. Compounding this challenge is the inherently slow pace of resistance breeding in conifers, constrained by long generation times, complex trait architectures, and extended field evaluation cycles [53,54]. This mismatch between rapid pathogen spread and slow breeding progress underscores the need for approaches that can substantially shorten screening and validation timelines.

Despite promising advances, several challenges remain in translating this pipeline from laboratory to field. Earlier studies using micropropagated pine shoots co-cultured with PWN demonstrated the value of clonal material for controlled host–pathogen studies [55]. Our pipeline builds on this foundation, formalizing it into a structured, tiered workflow for high-throughput screening, validation, propagation, and breeding. To realize its full potential, both translational bottlenecks and emerging technological synergies must be addressed.

### 6.1. Translational Challenges: From Laboratory to Field

In Vitro–Field Correlation: The ultimate validation of resistance is field performance under natural epidemic conditions. While the field transfer of *P. thunbergii* somatic plants is encouraging [56], future work must include long-term (ideally multi-year), multi-site trials of all in vitro-selected resistant clones to confirm the durability and stability of their resistance. Incorporating epidemiological modeling and risk analysis, as applied regionally for PWD [10], could enhance the predictive value of such trials.

Rooting and Acclimatization: Efficient rooting and acclimatization remain bottlenecks for many conifers [57]. The integration of beneficial ECM fungi is a promising strategy to overcome this hurdle [58,59,60]. We have demonstrated that inoculation with *P. orientalis* significantly improves acclimatization and resistance in *P. massoniana* somatic plantlets [43], and this approach holds similar promise for organogenesis-derived plantlets (Tier 2). This synergy between ECM fungi and in vitro-selected genotypes represents a highly promising integrated strategy. Future studies should define compatible mycorrhizal consortia and optimize cultivation protocols tailored to different species and genotypes.

Genotype Dependence: The efficiency of both organogenesis and somatic embryogenesis can be highly genotype-dependent [61,62]. Expanding the repertoire of responsive genotypes is an ongoing endeavor. Systematic comparison of culture responses among families and provenances, coupled with statistical designs to disentangle genetic from culture effects, will be essential to avoid unintended narrowing of the genetic base.

### 6.2. Technological Synergies and Future Directions

Recent advances in molecular studies of conifer–PWN interactions have underscored the complexity of resistance mechanisms, providing an important biological context for resistance breeding [63]. Integrating this framework with modern biological tools offers promising avenues for transformative gains in breeding speed, precision, and mechanistic understanding.

Multi-Omics Integration: Complementing transcriptomics with proteomics and metabolomics in resistant and susceptible lines [56] will elucidate molecular resistance networks, identify key regulators, and support biomarker-assisted selection. Recent proteomic work has already highlighted candidate proteins and pathways associated with PWN virulence and pine defense [11], providing concrete targets for functional validation. The genes and pathways revealed by these multi-omics analyses will generate a high-priority target list for downstream genetic engineering. The feasibility of this rational design approach is now supported by chromosome-level reference genomes for key pine species, including the susceptible *P. tabuliformis* [64], the primary breeding target *P. massoniana* [65], and the model conifer *P. taeda* [66]. Our pipeline directly leverages these resources. For instance, comparative transcriptomic data from resistant and susceptible genotypes can be analyzed within the genomic context of *P. massoniana* to pinpoint high-confidence candidate genes, such as those encoding putative susceptibility factors or central defense regulators. This integration forms the critical bridge between omics discovery and precision genetic engineering.

Gene Editing and Rational Design Breeding: The embryogenic cell lines central to Tier 3 serve as ideal recipients for genetic manipulation. We have established efficient *Agrobacterium*-mediated transformation for embryogenic cultures of *P. massoniana* and related pines (e.g., *P. elliottii*). This transformation platform is integrated with the high-throughput phenotyping capability of Tier 1, creating a functional loop for the initial assessment of nematode resistance in engineered lines. This integrated “gene introduction, somatic embryo regeneration, and early-stage resistance screening” pipeline establishes the critical infrastructure required for precise genome editing. Building on this platform, CRISPR-Cas-based technologies represent the key opportunity to upgrade the pipeline from a “screening platform” to a “design platform” [67]. Susceptibility genes or core defense regulators identified via multi-omics can be targeted for editing. Edited embryogenic cell lines could be rapidly validated in Tier 1, regenerated via somatic embryogenesis (Tier 3), and advanced to Tier 2 for whole-plant evaluation. This creates a closed-loop strategy that entails target identification, followed by precision editing, and culminates in multi-tier phenotypic validation. The feasibility of this approach is supported by advances in forest-tree gene editing, including strategies to recover transgene-free edited plants, as demonstrated in poplar [68]. For example, our prior transcriptomics of *P. massoniana* responding to PWN identified strongly induced defense genes, including the pathogenesis-related protein PR1 [69], a classic marker of systemic acquired resistance. This candidate can be validated across backgrounds within our pipeline and then precisely engineered—for instance, via CRISPR-Cas9-mediated promoter editing—to enhance its expression in a susceptible line. The engineered lines can be rapidly screened for improved PWN resistance in Tier 1. Looking ahead, the successful deployment of gene-edited, resistant pines will also depend on addressing regulatory frameworks and fostering public understanding and acceptance of this technology in forestry.

Advanced in Vitro Selection: Incorporating pathogen-derived elicitors or culture filtrates into the culture medium could intensify selection pressure at the cellular level (Tier 1), potentially revealing cryptic resistance alleles [70]. Coupling such refined selection with high-content imaging and automated phenotyping would further increase throughput and objectivity, making the pipeline more amenable to large breeding populations.

Ultimately, the integration of these technologies will transform the framework into a dynamic, community-accessible model system. Its closed-loop, knowledge-driven design ensures that data from multi-omics, gene editing, and advanced phenotyping continuously refine both the platform and our understanding of PWD. This iterative process embodies the type of shared experimental system needed to accelerate discovery across forest pathosystems [14].

### 6.3. Advantages and Limitations of In Vitro–Based Resistance Screening

Conventional resistance breeding in conifers is constrained by long generation times and delayed phenotypic evaluation. In contrast, the proposed three-tier in vitro pipeline enables early-stage resistance assessment under controlled conditions, thereby streamlining early-stage resistance evaluation and reducing the field-testing burden.

Despite these advantages, several limitations should be acknowledged. The scalability of in vitro systems can be constrained by genotype dependence and labor requirements, and prolonged tissue culture may pose a risk of somaclonal variation. Recognizing these challenges is essential for the responsible application of in vitro screening approaches within operational breeding programs.

## 7. Conclusions

In conclusion, we present a three-tier, dual-output strategy that bridges the in vitro–field gap in managing pine wilt disease. By integrating high-throughput cellular screening, whole-plant validation, and scalable production into a closed-loop, knowledge-driven workflow, this approach accelerates the development of resistant germplasm while supporting mechanistic research. Validated across pine species, this strategy offers a practical and replicable framework to address the “time conflict” in perennial crop breeding, contributing to the development of more resilient forests.

## Figures and Tables

**Table 1:** Cross-species validation of the Three-Tiered Framework for breeding PWN-resistant pines.

Species/ Application	Tier 1: Callus Screening	Tier 2: Organogenesis Validation	Tier 3: Somatic Embryogenesis & Beyond	Key Resistant Materials/Main Findings
*P. massoniana*	Established and used for screening [24]	Established and used for screening [40]	Established; Enables mycorrhization & enhanced performance [43]	Callus: GX20-3-3; Microshoot: Clone 227; Somatic: ECL 20-1-7
*P. densiflora*	Established the system [23]	Established and used for screening [19,20,37]	Not Reported	Microshoot: Clone 8–4

## Data Availability

No new data were created in this study.
